# Supplementary material for: Measuring, visualizing and diagnosing reference bias with biastools
Source: bioRxiv. 2024 Feb 15:2023.09.13.557552. Originally published 2023 Sep 16. Preprint. [Version 2] doi: 10.1101/2023.09.13.557552 (PMC10515925; doi:10.1101/2023.09.13.557552)
Supplement: Supplement 1 [file NIHPP2023.09.13.557552v2-supplement-1.pdf]

bioRxiv preprint doi: https://doi.org/10.1101/2023.09.13.557552; this version posted February 15, 2024. The copyright holder for this preprint (which was not certified by peer review) is the author/funder, who has granted bioRxiv a license to display the preprint in perpetuity. It is made available under aCC-BY 4.0 International license.

Supplementary Material

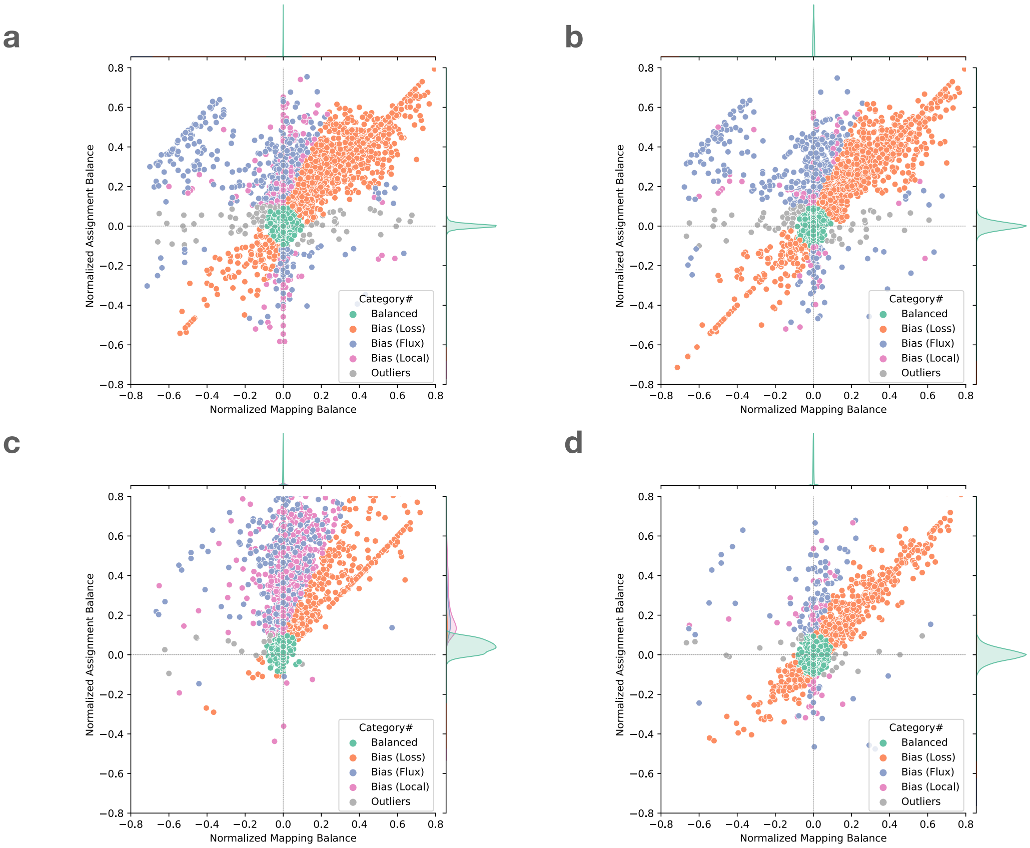

Figure S1: Normalized mapping balance to normalized assignment balance (NMB-NAB) plot of **a.** SNV sites with naive assignment method, **b.** SNV sites with context-aware assignment method, **c.** insertion and deletion sites with naive assignment method, and **d.** insertion and deletion sites with context-aware assignment method. All variants are listed in the figure.

bioRxiv preprint doi: https://doi.org/10.1101/2023.09.13.557552; this version posted February 15, 2024. The copyright holder for this preprint (which was not certified by peer review) is the author/funder, who has granted bioRxiv a license to display the preprint in perpetuity. It is made available under aCC-BY 4.0 International license.

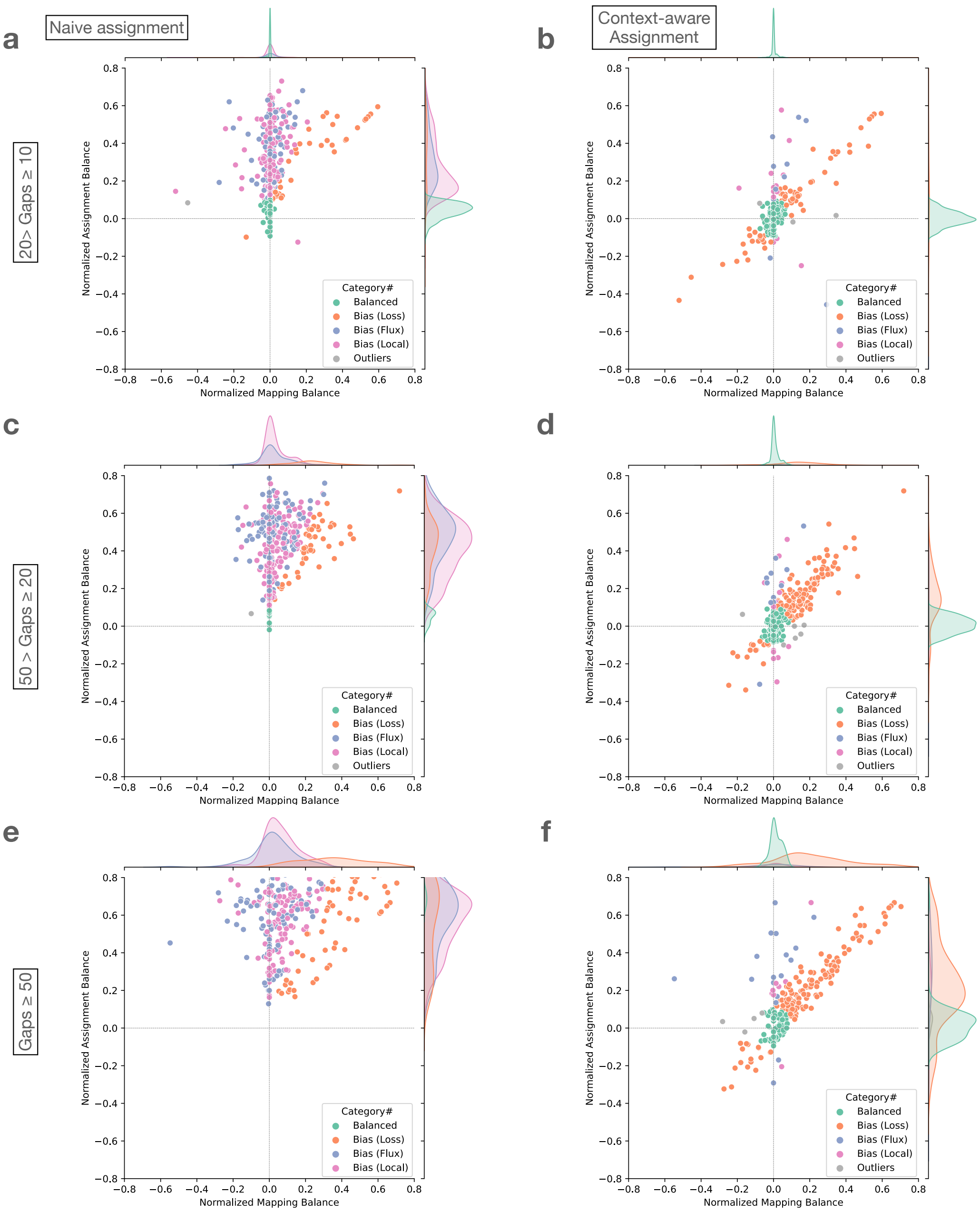

Figure S2: Normalized mapping balance to normalized assignment balance (NMB-NAB) plot from Bowtie 2 mapping simulated reads with naive assignment method on **a.** gaps between 10 and 20 bp, **c.** gaps between 20 and 50 bp, and **e.** gaps greater or equal to 50 bp. And the same dataset assigned by context-aware method on **b.** gaps between 10 and 20 bp, **d.** gaps between 20 and 50 bp, and **f.** gaps greater or equal to 50 bp.

bioRxiv preprint doi: https://doi.org/10.1101/2023.09.13.557552; this version posted February 15, 2024. The copyright holder for this preprint (which was not certified by peer review) is the author/funder, who has granted bioRxiv a license to display the preprint in perpetuity. It is made available under aCC-BY 4.0 International license.

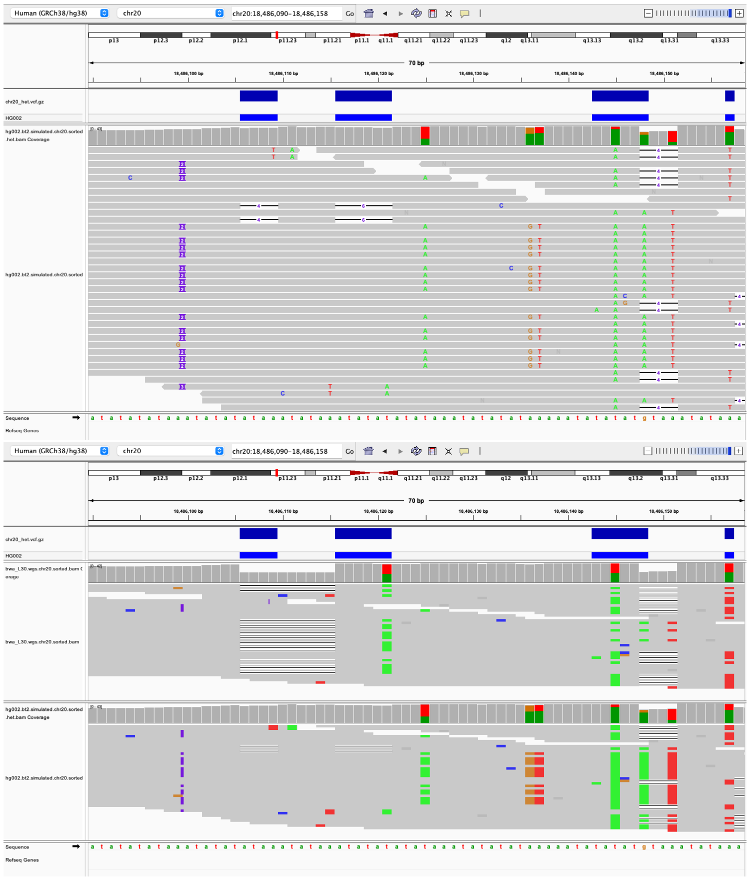

Figure S3: Local decision of consecutive three deletions in a repetitive region. Upper: Bowtie2's local alignment decision. In many alignments, the two deletions on the left are not evident, and instead a 2 bp insertion is introduced to the left of the deletions. This creates a shift in reference coordinates that prevents biastools' context-aware assignment method from assigning the reads correctly. Lower: The different local alignment decisions made by BWA MEM and Bowtie2 using the same set of the reads.

bioRxiv preprint doi: https://doi.org/10.1101/2023.09.13.557552; this version posted February 15, 2024. The copyright holder for this preprint (which was not certified by peer review) is the author/funder, who has granted bioRxiv a license to display the preprint in perpetuity. It is made available under aCC-BY 4.0 International license.

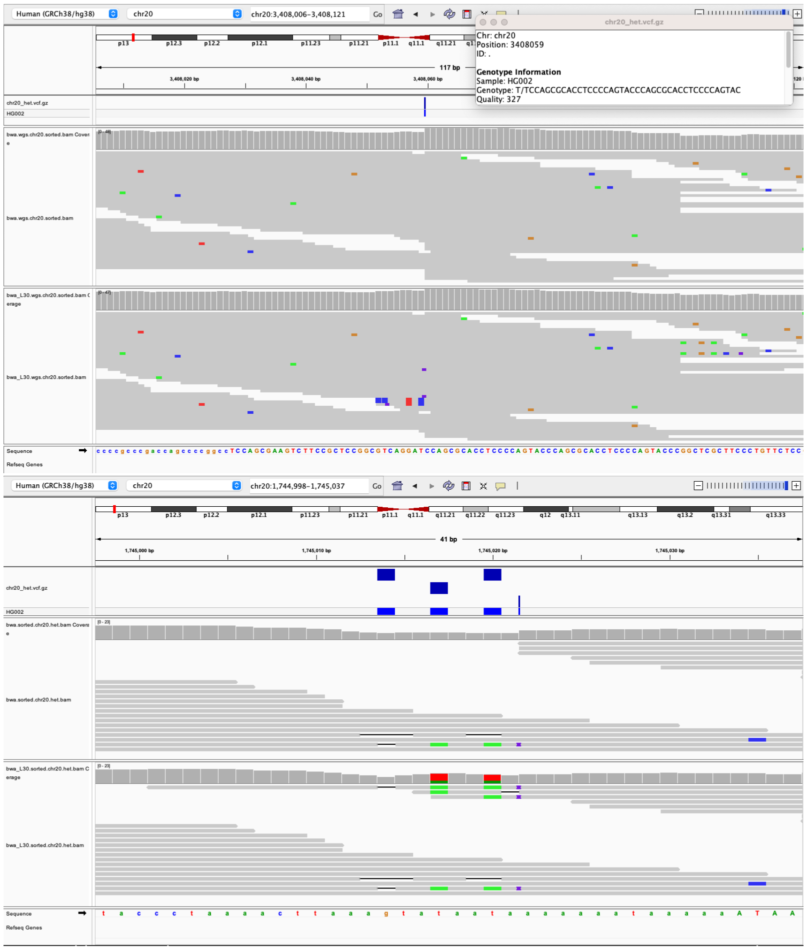

Figure S4: In the IGV, the upper track is the BWA MEM default setup while the lower track is the BWA MEM with clipping penalty set to 30 instead of default 5. All the reads contain the 39 bp insertion are clipped in the default BWA MEM setup, thus create the biases toward the ALT allele. The BWA MEM with -L 30 behaves more like an end-to-end aligner, thus can handle the insertion without clipping.

bioRxiv preprint doi: https://doi.org/10.1101/2023.09.13.557552; this version posted February 15, 2024. The copyright holder for this preprint (which was not certified by peer review) is the author/funder, who has granted bioRxiv a license to display the preprint in perpetuity. It is made available under aCC-BY 4.0 International license.

Table S1: Number of balanced sites and different categories of biased sites on chromosome 16. The simulated WGS reads of HG002 are aligned by 8 different tools. The best results of balanced and Bias "Loss" are marked in bold and underline. The second best results are marked in bold.

		Bowtie 2	BWA-MEM	BWA-MEM (-L 30)	Minimap2	Giraffe-linear	Giraffe-major	Giraffe-pop5	Giraffe-1KGP
SNV	Balanced	69847	69824	70073	69855	70032	70034	**70106**	**70102**
	Bias (Loss)	3369	3306	3060	3291	3129	3104	**3008**	**2980**
	Bias (Flux)	**963**	1047	1054	1035	**981**	984	989	1013
	Bias (Local)	**139**	167	166	**164**	185	211	229	234
	Outliers	138	112	**103**	**111**	129	123	124	127
Gap	Balanced	**14061**	13723	**14229**	13774	13952	13989	14007	14046
	Bias (Loss)	1151	1319	**878**	1291	1155	1113	1083	**1052**
	Bias (Flux)	**212**	287	278	274	252	249	**245**	247
	Bias (Local)	**136**	223	**167**	212	194	202	221	214
	Outliers	**31**	39	39	40	38	38	35	**32**

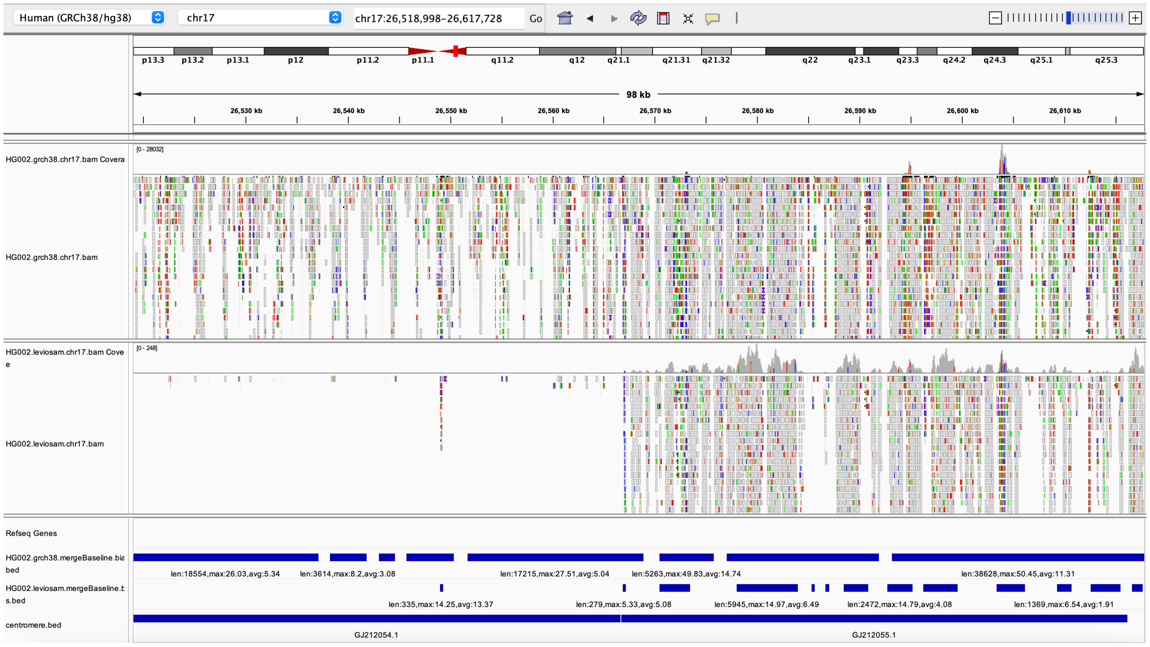

Figure S5: An example of the low coverage result of LevioSAM 2 and direct-to-GRC methods. The top two panels show how the reads aligned by the two methods. The bottom three panels show the biased regions called by `biastools scan` in direct-to-GRC, LevioSAM 2, and the range of the centromeric reigon. While LevioSAM 2 did not classify the region on the left as biased, this is due simply to the low coverage in that region.
