# Supplementary material for: Measuring, visualizing and diagnosing reference bias with biastools
Source: bioRxiv. 2023 Sep 16:2023.09.13.557552. Preprint. [Version 1] doi: 10.1101/2023.09.13.557552 (PMC10515925; doi:10.1101/2023.09.13.557552)
Supplement: Supplementary file 1 [file media-1.pdf]

Supplementary Material

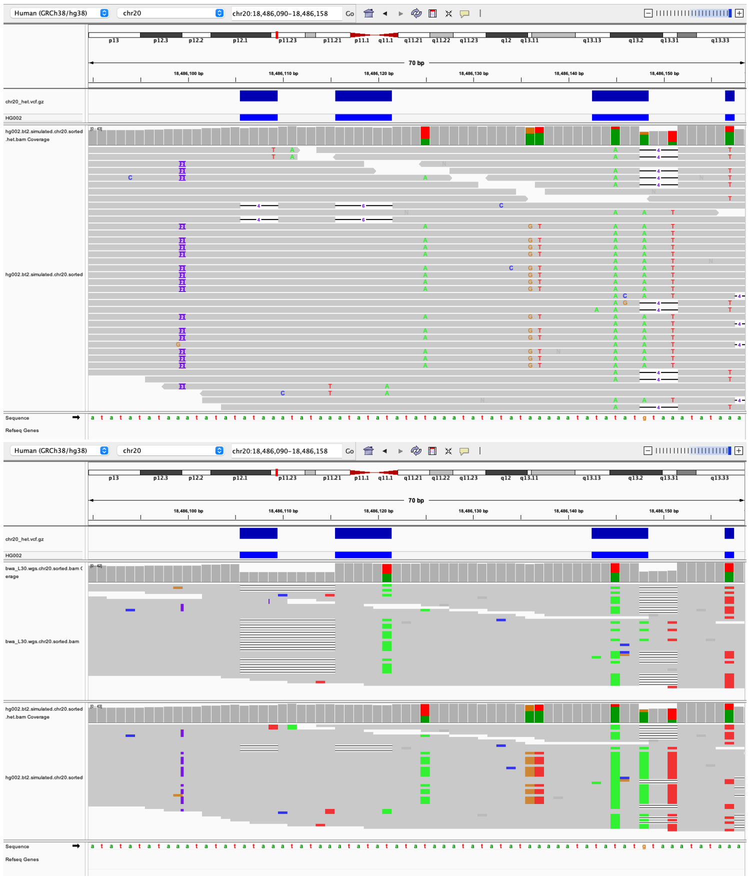

Figure S1: Local decision of consecutive three deletions in a repetitive region. Upper: Bowtie2's local alignment decision. In many alignments, the two deletions on the left are not evident, and instead a 2 bp insertion is introduced to the left of the deletions. This creates a shift in reference coordinates that prevents biastools' context-aware assignment method from assigning the reads correctly. Lower: The different local alignment decisions made by BWA MEM and Bowtie2 using the same set of the reads.

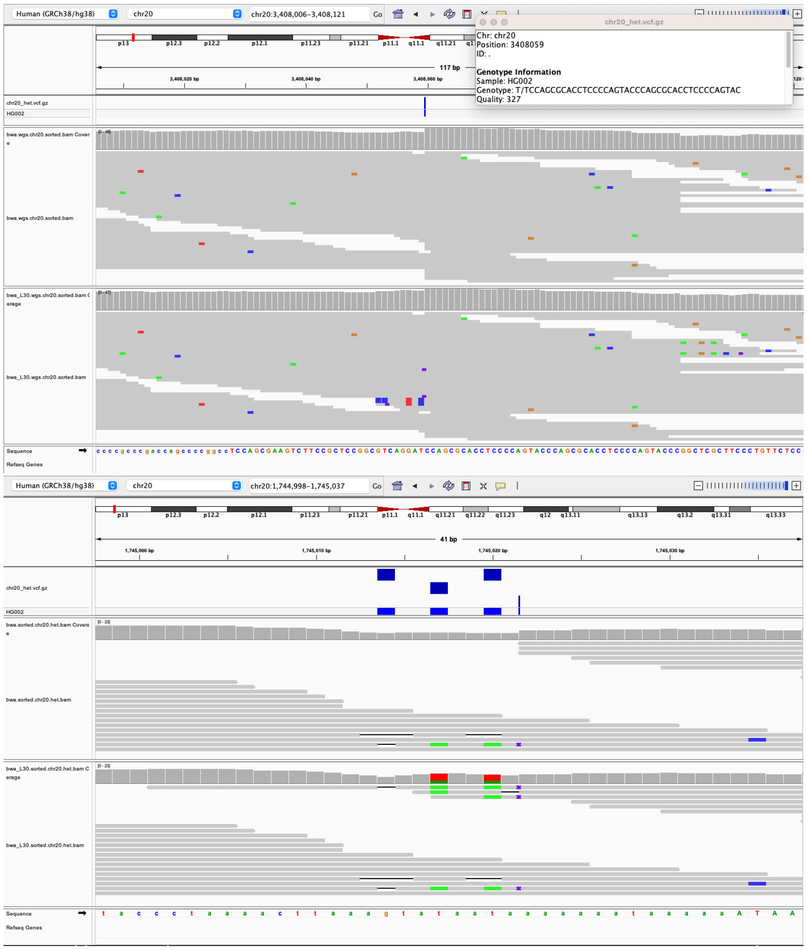

Figure S2: In the IGV, the upper track is the BWA MEM default setup while the lower track is the BWA MEM with clipping penalty set to 30 instead of default 5. All the reads contain the 39 bp insertion are clipped in the default BWA MEM setup, thus create the biases toward the ALT allele. The BWA MEM with -L 30 behaves more like an end-to-end aligner, thus can handle the insertion without clipping.

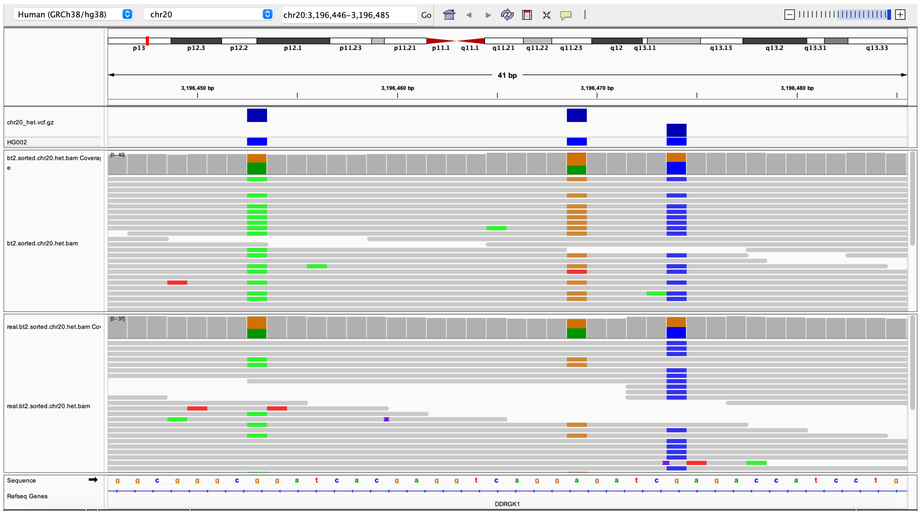

Figure S3: An example of how bias measurements can be complicated by the lack of phasing information in a VCF file. This example uses the HG002 GIAB VCF file which lacks phasing. The upper track shows the pileup for reads simulated from the VCF file. Lower track: the pule for real HG002 reads. Notice the phasing disagreement between the two pileups visible for the second (orange coloring) and third (blue coloring) variants.

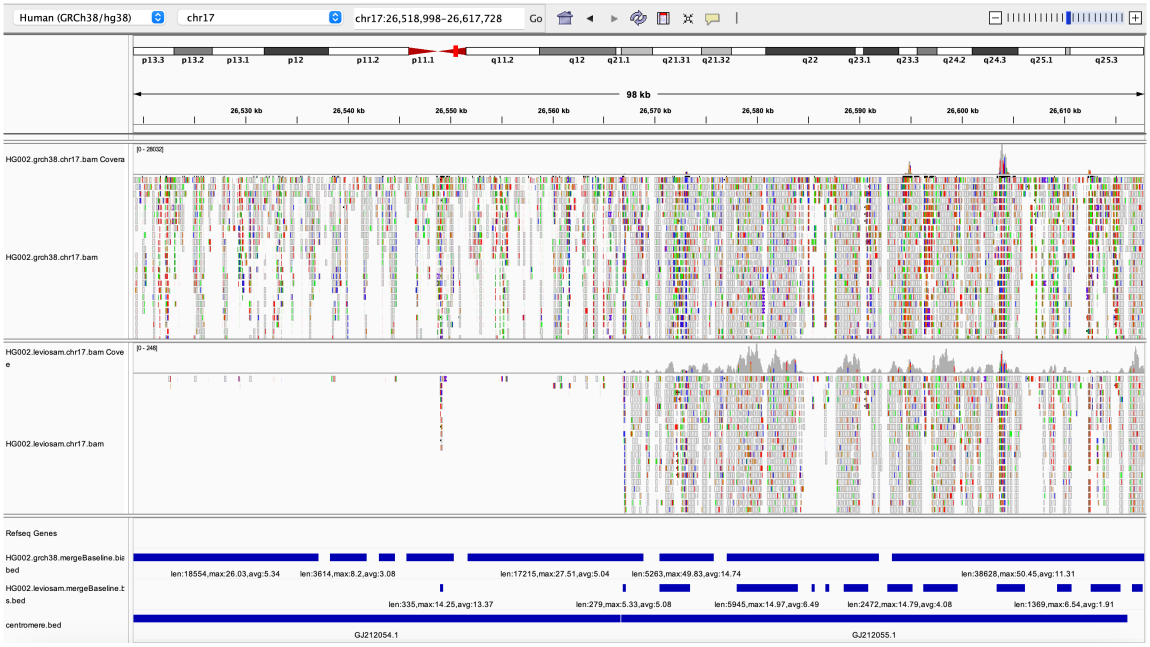

Figure S4: An example of the low coverage result of LevioSAM 2 and direct-to-GRC methods. The top two panels show how the reads aligned by the two methods. The bottom three panels show the biased regions called by `biastools scan` in direct-to-GRC, LevioSAM 2, and the range of the centromeric reigon. While LevioSAM 2 did not classify the region on the left as biased, this is due simply to the low coverage in that region.
